# KD-SSGD: knowledge distillation-enhanced semi-supervised germination detection

**DOI:** 10.3389/fpls.2025.1688792

**Published:** 2025-12-08

**Authors:** Chengcheng Chen, Di Luo, Tiantian Pang, Xianchang Wang, Ronghao Fu, Kaiyao Gou, Helong Yu

**Affiliations:** 1 School of Computer Science, Shenyang Aerospace University, Shenyang, China; 2 College of Computer Science and Technology, Jilin University, Changchun, China; 3 College of Information Technology, Jilin Agricultural University, Changchun, China

**Keywords:** semi-supervised object detection, knowledge distillation, germination detection, ensemble learning, deep learning

## Abstract

With the rapid development of precision agriculture, seed germination detection is crucial for crop monitoring and variety selection. Existing fully supervised detection methods rely on large-scale annotated datasets, which are costly and time-intensive to obtain in agricultural scenarios. To tackle this issue, we introduce a knowledge distillation–enhanced semi-supervised germination detection framework (KD-SSGD) that requires no pre-trained teacher and supports end-to-end training. Built on a teacher–student architecture, KD-SSGD introduces a lightweight distilled student branch and three key modules: Weighted Boxes Fusion (WBF) to optimize pseudo-label localization, Feature Distillation Loss (FDL) for deep semantic knowledge transfer, and Branch-Adaptive Weighting (BAW) to stabilize multi-branch training. On the Maize-Germ (MG) open-access dataset, KD-SSGD achieves 47.0% mAP with only 1% labeled data, outperforming Faster R-CNN (35.6%), Mean Teacher (41.9%), Soft Teacher (45.1%), and Dense Teacher (45.0%), and reaches 59.3%, 62.8%, and 65.1% mAP at 2%, 5%, and 10% labeled ratios. On the Three Grain Crop (TGC) open-access dataset, which achieves 73.3%, 75.3%, 75.6%, and 76.1% mAP at 1%, 2%, 5%, and 10% labels, surpassing mainstream semi-supervised methods and demonstrating robust cross-crop generalization. The results indicate that KD-SSGD could generate high-quality pseudo-labels, effectively transfer deep knowledge, and achieve stable high-precision detection under limited supervision, providing an efficient and scalable solution for intelligent agricultural perception.

## 1 Introduction

Recent advances in deep learning have made object detection a central task in modern computer vision research (Wu et al., 2020; Carion et al., 2020). Current detection approaches have achieved remarkable performance through diverse architectures, including one-stage models (e.g., RetinaNet (Lin et al., 2017b), FCOS (Tian et al., 2019)), two-stage or cascaded methods (e.g., Faster R-CNN (Ren et al., 2017), Cascade R-CNN (Cai and Vasconcelos, 2021)), and Transformer-based frameworks (e.g., Deformable DETR (Zhu et al., 2021), DAB-DETR (Liu et al., 2022)). Multi-scale feature enhancement modules, for example the Feature Pyramid Network (FPN) (Lin et al., 2017a), have significantly strengthened detection performance over different object scales. However, the effectiveness of these methods is highly contingent upon comprehensive, accurately labeled datasets, whose collection is costly, particularly in domain-specific fields such as agriculture.

Seed germination detection exemplifies these challenges. Accurate identification of germination states is critical for assessing seed vigor and supporting intelligent breeding (Genze et al., 2020; Nehoshtan et al., 2021). This task requires recognizing fine-grained features—such as radicle length—from image sequences, often under challenging conditions involving small object size, ambiguous boundaries, occlusion, and severe class imbalance. These factors limit the effectiveness of conventional supervised methods in meeting the high-throughput and low-cost demands of modern agricultural applications.

To reduce reliance on labeled data, semi-supervised approaches have received increasing focus in object detection. Semi-supervised object detection (SSOD) (Chen L. et al., 2024; Fu et al., 2024a, b)commonly employs a teacher–student framework, where the supervisory signals generated from the teacher network are used to train the student branch. In agricultural applications, several studies have explored SSOD. Tseng et al (Tseng et al., 2023). constructed the smallSSD dataset and released a Python package. They further proposed a calibrated teacher–student framework that integrated data augmentation, weak teacher ensembles, and class-wise dynamic calibration to improve pseudo-label quality and robustness in agricultural environments. Zhou et al (Zhou and Shi, 2025). introduced the Perceptive Teacher framework to address small-object detection in Brassica seedlings and pest monitoring, using difficulty-adaptive learning and center distance matching, which significantly improved pseudo-label accuracy and stability under limited annotations. Yang et al (Yang et al., 2025a). combined UAV imagery with SSL for early maize seedling monitoring, constructing multi-temporal field image datasets and employing Mosaic augmentation, a pseudo-label allocator (PLA), and differentiated regression and classification losses to improve detection of critical targets. Despite progress achieved by existing methods, seed germination detection still faces challenges such as minute root tips, blurred boundaries, occlusions, and imbalanced data distributions. STAC (Sohn et al., 2020b) pioneered consistency training but relied on separate training, limiting its performance. Mean Teacher (Tarvainen and Valpola, 2017) improves stability by smoothing teacher model updates via exponential moving average (EMA), yet its heavy reliance on student predictions is prone to error accumulation and confirmation bias. Subsequent approaches are mostly variants; for instance, Soft Teacher (Xu et al., 2021) designs specific modules for two-stage detection architectures (e.g., Faster R-CNN) to refine label quality and boost performance. Dense Teacher (Zhou et al., 2022) improves dense pseudo-labeling for anchor-based detectors but remains sensitive to pseudo-label noise and lacks generalization to diverse architectures. However, such improvements lack generalizability and are difficult to transfer across different detector frameworks.

Knowledge distillation (KD) (Hinton et al., 2015; Gou et al., 2021) conveys information between a high-capacity teacher network and a compact student through soft targets, feature representations, or attention cues, thereby enhancing deployment efficiency. In agricultural computer vision, KD has been successfully applied: WD-YOLO (Yang et al., 2025) used a YOLOv10l teacher to train a lightweight student, achieving 65.4% mAP@0.5 while reducing parameters; MobileNetV3-based weeding navigation (Gong et al., 2024) incorporated CBAM attention and distilled teacher knowledge to the student, reaching 92.2% mAP at 39 FPS; hybrid ViT distillation (Mugisha et al., 2025) transferred both logits and spatial attention from a Swin Transformer teacher to MobileNetV3, achieving 92.4% accuracy with only 0.22 GFLOPs and 13 MB parameters. Most KD methods, however, rely on a two-stage training process with pre-trained teachers, which is data- and time-intensive, limiting applicability in annotation-scarce agricultural settings. To address the challenges of data dependency, error accumulation in semi-supervised Mean Teacher frameworks under extremely low annotation conditions, and the reliance of most knowledge distillation (KD) methods on pre-trained teacher models and two-stage training, while being inspired by the concept of ensemble learning Guo and Gould (2015), we propose the KD-SSGD framework. This paper presents the following major contributions:

• End-to-end knowledge distillation-based semi-supervised detection: KD-SSGD combines pseudo label learning and knowledge distillation in a cohesive end-to-end framework, extending the application of knowledge distillation in semi-supervised detection.

• Three collaborative mechanisms to enhance detection performance: In germination detection tasks, WBF improves pseudo-label localization accuracy, FDL enables multi-level knowledge transfer of fine-grained radicle and shoot features, and BAW balances multi-branch losses while stabilizing training, collectively boosting overall detection performance.

• Multi-crop germination detection under extremely low annotation conditions: KD-SSGD accurately detects germination targets across different developmental stages of multiple crops with very limited labeled data, consistently outperforming conventional fully supervised methods and remaining competitive with mainstream semi-supervised approaches, while demonstrating robustness and high reliability.

The subsequent sections are arranged as follows: Section 2 details the proposed methodology, including dataset partitioning and description, evaluation metrics, method overview, Weighted Box Fusion, Feature Distillation Loss, Branch Adaptive Weighting, and implementation details. Section 3 comprehensively details the experimental results and analysis, including detection performance across varying labeling ratios and critical insights from ablation studies. Concluding in Section 4, the paper discusses promising research avenues for future work.

## 2 Materials and methods

### 2.1 Dataset overview and split strategy

This research conducts a comprehensive assessment of the proposed methodology using both the publicly available Maize-Germ (MG) RGB image dataset (Chen C. et al., 2024) and the Three Grain Crop (TGC) dataset (Genze et al., 2020), focusing on seed germination detection. The MG dataset comprises five annotated categories: ungerminated, germinating, germinated, primary root, and secondary root, which collectively represent the full spectrum of maize germination stages. The TGC dataset captures the germination process of three grain crops: Pennisetum glaucum (PG), Secale cereale (SC), and Zea mays (ZM), with each crop categorized into germinated and ungerminated classes.To assess the generalization capability of the proposed method, the three crop-specific subsets, each containing germinated and ungerminated classes, were combined into a single dataset comprising six labeled categories. For experimental setup, 80% of the MG dataset was used for training and 20% for validation through random splitting, while the TGC datasets followed the official training, validation, and test splits, but the validation and test sets were merged for a more comprehensive evaluation, as the original test set generally yielded higher performance. All model hyperparameter tuning and performance evaluation were conducted on independent validation sets to ensure fairness and reproducibility. Regarding data partitioning, following the semi-supervised experimental protocol proposed in MixPL (Chen et al., 2023), each training set was subsequently partitioned into labeled and unlabeled portions. Specifically, for the MG dataset (**Table 1**), 1%, 2%, 5%, and 10% of the train dataset were randomly chosen as labeled data, with the remainder used as unlabeled data to simulate a realistic scenario of limited annotation. For the merged TGC dataset (**Table 2**), the same labeled proportions were adopted, thereby systematically validating the generalization and stability of the proposed method across different crop types and data distributions.

**Table 1:** Data partition and instance counts of the MG dataset at different labeled ratios.

Data	Labeled	Images	Ungerm.	Germin.	Germ.	Pri. root	Sec. root	Total
Training set	–	14500	119731	5660	1564	4101	13944	145000
Validation set	–	3625	30111	1382	372	986	3399	36250
Semi-supervised	1%	143	1220	67	12	21	110	1430
	2%	286	2393	135	31	66	235	2860
	5%	703	5852	281	61	175	661	7030
	10%	1363	11271	546	151	354	1308	13630

“Ungerm.”, ungerminated seeds; “Germin.”, germinating seeds; “Germ.”, germinated seeds; “Pri. root”, primary root; “Sec. root”, secondary root.

Semi-supervised rows indicate training subsets for labeled ratios.

**Table 2:** Image and instance statistics of the merged TGC dataset with labeled ratios.

Data	Labeled	Images	PG_im	PG_el	SC_im	SC_el	ZM_im	ZM_el	Total
Training set	–	18884	24883	38555	28581	32219	46216	16931	187385
Validation set	–	4913	9655	6835	8144	8103	10775	5036	48548
Semi-supervised	1%	184	399	225	271	319	170	440	1824
	2%	367	746	489	501	679	378	847	3640
	5%	910	1846	1212	1308	1622	844	2184	9016
	10%	1774	3503	2453	2572	3148	1516	4401	17593

PG (*Pennisetum glaucum*), SC (*Secale cereale*), ZM (*Zea mays*); “ im”, ungerminated seeds; “ el”, germinated seeds. Semi-supervised rows indicate training subsets for labeled ratios.

### 2.2 Evaluation metrics

**Figure 1:**
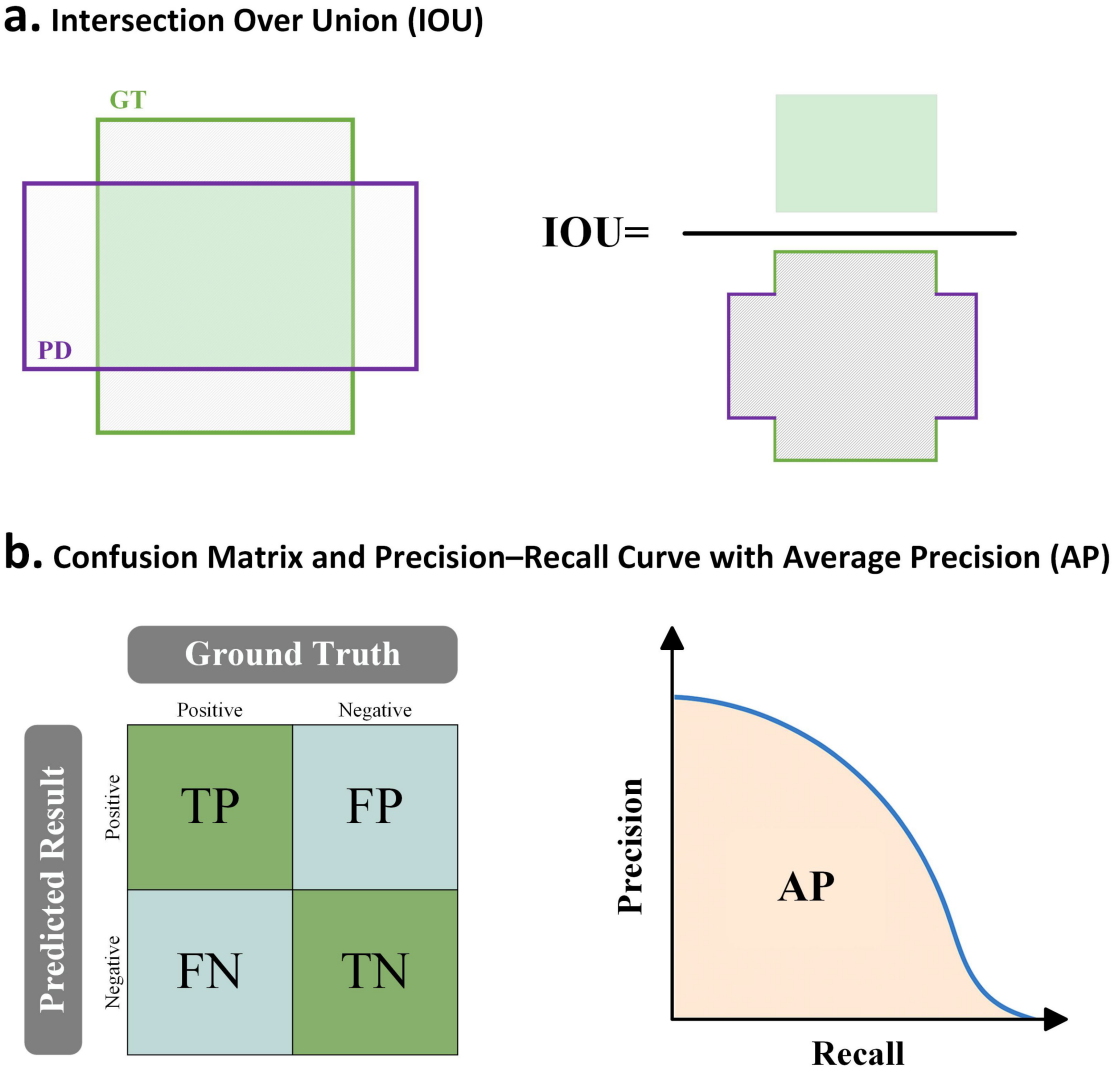
Key components of model performance evaluation. **(A)** Intersection over Union (IOU) is defined as the ratio of the overlapping area between the predicted box (PD, purple) and the ground truth box (GT, green) to their union. **(B)** The left panel shows the confusion matrix, representing the correspondence between predicted and actual labels, including TP, FP, FN, and TN, which are used to compute precision and recall. The right panel presents the precision–recall curve, with average precision (AP) corresponding to the area under the curve (orange shaded region).

For a thorough assessment of the trained models’ detection capabilities, this study primarily uses the Average Precision (AP) as its key metric (Lin et al., 2014). The AP calculation relies on the Intersection over Union (IoU) between a predicted bounding box and its corresponding ground-truth box. A prediction is categorized as a true positive (TP) when its IoU score surpasses a predetermined threshold and its class label is correct. As demonstrated in **Figure 1a**, IoU quantifies the accuracy of localization by representing the proportion of the overlapping region to the total region of the combined boxes.

Precision and Recall are used to evaluate seed germination detection performance. Precision represents the proportion of correctly predicted germinated seeds (TP) among all seeds predicted as germinated (TP + FP), reflecting prediction accuracy. Recall denotes the proportion of actual germinated seeds correctly detected (TP) among all ground-truth germinated seeds (TP + FN), indicating the model’s ability to identify positive instances. The definitions and relationships of TP, TN, FP, and FN are illustrated in a confusion matrix shown in **Figure 1b** (left panel). **Figure 1b** (right panel) shows the Precision–Recall (PR) curve, with AP defined as the interpolated area under the curve. For multi-class tasks, AP is computed per class and then averaged to obtain the mean Average Precision. The standard COCO metrics are reported: mAP@[0.50:0.95] (mean of class-averaged AP values at IoU thresholds from 0.50 to 0.95 in steps of 0.05), as well as mAP@0.50 and mAP@0.75, corresponding to class-averaged AP at fixed IoU thresholds of 0.50 and 0.75, respectively. In the following results, mAP@[0.50:0.95] is simply referred to as mAP.

### 2.3 Method overview

**Figure 2:**
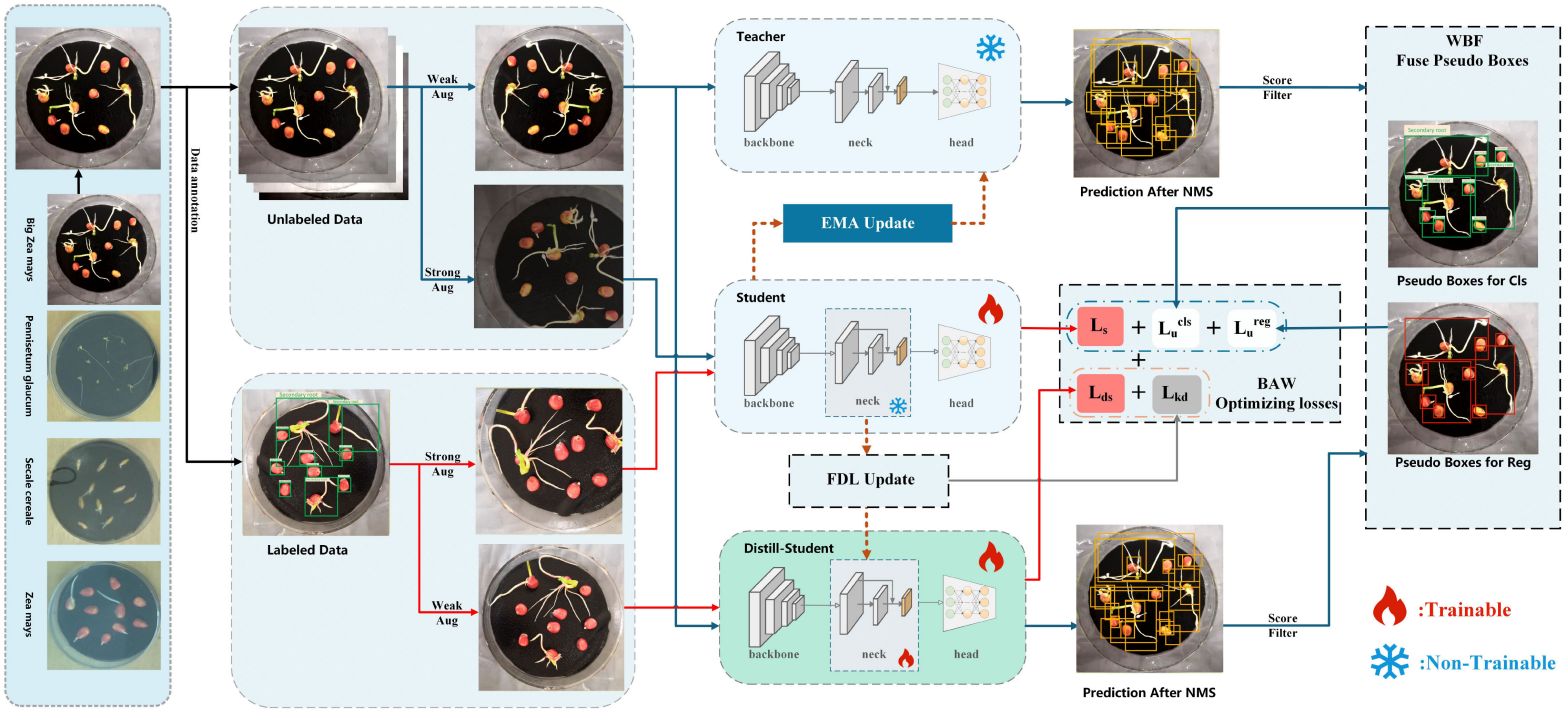
Overview of the proposed KD-SSGD method. The model adopts a teacher–student architecture with an additional lightweight distilled student branch. During each training iteration, mixed batches containing both labeled and unlabeled images are sampled. The teacher and distilled student jointly generate predictions on unlabeled data, which are fused via Weighted Boxes Fusion (WBF) to produce robust pseudo-labels. These pseudo-labels, along with ground-truth labels, supervise the student model. Feature Distillation Loss (FDL) enables the distilled student to learn intermediate representations from the student model, while Branch-Adaptive Weighting (BAW) dynamically balances all loss terms to ensure stable training and optimal performance.

This paper proposes the KD-SSGD framework, which is built upon the classical teacher–student architecture and incorporates ensemble learning to fully exploit multi-model predictions (see **Figure 2**). We introduce a lightweight distilled student branch that adopts the same architecture as the student model but with a simplified backbone. The teacher model and the distilled student jointly perform inference on unlabeled images, and their predictions are first fused using WBF to generate more reliable pseudo-labels, thereby improving the semi-supervised training of germination detection in agricultural seed phenotyping. Meanwhile, the distilled student aligns its feature representations with the student model via FDL, which enhances feature expressiveness. In the multi-branch joint optimization process, we further incorporate the BAW mechanism to dynamically balance the losses across different branches, effectively improving training stability and overall performance.

In each training iteration, we construct mixed batches containing both labeled and unlabeled samples according to a predefined ratio. Both the teacher and the distilled student models perform inference on the unlabeled data, and their outputs are combined via WBF to produce reliable pseudo-labels. The generated pseudo-labels are subsequently employed to guide the training of the student model on unlabeled data, thereby improving detection accuracy. In addition, we adopt the dual-branch augmentation paradigm from FixMatch (Sohn et al., 2020a) to enhance generalization: in the unsupervised branch, the teacher model applies weak augmentation to ensure pseudo-label stability, while the student model applies strong augmentation to encourage learning of perturbation-invariant features; in the supervised branch, the student model uses strong augmentation to fully exploit the labeled data, whereas the distilled student applies milder augmentation to maintain training stability. Meanwhile, the distilled student is further optimized through FDL, which aligns its intermediate representations with those of the student model, thereby enhancing its performance and improving the expressiveness of pseudo-labels.To coordinate optimization across branches, BAW mechanism dynamically adjusts loss weights, improving convergence stability and overall performance. The combined loss function is formulated as:

(1) $$L=\lambda_{1}L_{\text{sup}}^{s}+\lambda_{2}L_{\text{sup}}^{ds}+\alpha L_{\text{unsup}}+\beta L_{\text{kd}}$$

where $L_{\text{sup}}^{s}$ is the supervised loss for the student model on labeled data, $L_{\text{sup}}^{ds}$ is the supervised loss for the distilled student model, $L_{\text{unsup}}$ is the unsupervised loss of the student model on unlabeled data guided by pseudo-labels, and $L_{\text{kd}}$ is the knowledge distillation loss between the student and distilled student models. The hyperparameters *λ*_1_*, λ*_2_*, α, β* are the respective weighting coefficients. The mathematical formulations of the individual loss components are specified in Equations 2–4.

(2) $$L_{\text{sup}}^{\left(\text{model}\right)}=\frac{1}{N_{l}}\sum_{i=1}^{N_{l}}(L_{\text{cls}}(I_{l}^{i})+L_{\text{reg}}(I_{l}^{i}))$$

(3) $$L_{\text{unsup}}=\frac{1}{N_{u}}\sum_{i=1}^{N_{u}}(L_{\text{cls}}(I_{u}^{i})+L_{\text{reg}}(I_{u}^{i}))$$

(4) $$L_{\text{kd}}=\frac{1}{N_{l}}\sum_{i=1}^{N_{l}}\text{KD}(f_{s}(I_{l}^{i})\;||\;f_{ds}(I_{l}^{i}))$$

where *model* refers to either the student (s) model or the distilled student (ds) model. $I_{l}^{i}\;$ and $I_{u}^{i}\;$ indicate the *i*-th labeled and unlabeled images, respectively. $L_{\text{cls}}$ and $L_{\text{reg}}$ represent classification and bounding box regression losses. *N_l_* and *N_u_* are the numbers of labeled and unlabeled samples. *f_s_*(·) and *f_ds_*(·) denote the feature mappings of the student and distilled student models, and KD(·||·) denotes the loss used for knowledge distillation.

Through a multi-branch loss, the roles of teacher, student, and distilled-student models are exploited collaboratively. On labeled images, both the student model and the distilled student model independently perform supervised learning based on Equation 2, ensuring adequate representation capacity. On unlabeled images, the student model is optimized through the unsupervised loss defined in Equation 3, guided by pseudo-labels. Simultaneously, the distilled student model learns structural knowledge from the student model via feature distillation formulated in Equation 4, thereby enhancing its semantic modeling capability. The weighting coefficients *λ*_1_*, λ*_2_*, α, β* are dynamically tuned by the BAW mechanism to ensure stability and optimal final performance.

To better elucidate the mechanism of each module in the framework, detailed descriptions of WBF, FDL, and BAW are provided in the following subsections.

### 2.4 Weighted boxes fusion

To effectively fuse detection results from the teacher and distilled student models, we adopt WBF method (Solovyev et al., 2021) to fuse predictions and produce reliable pseudo-labels. Unlike traditional Non-Maximum Suppression (NMS) (Neubeck and Van Gool, 2006) or Soft-NMS (Bodla et al., 2017), WBF does not discard overlapping boxes. Instead, it computes a confidence-weighted average of the bounding box coordinates, fully leveraging predictions from multiple models to produce more stable and accurate pseudo-labels.

Specifically, for model m, the set of predicted bounding boxes on a single image is formally defined in Equation 5:

(5) $$B^{\left(m\right)}={\left\{B_{i}^{\left(m\right)}=(x_{i}^{\left(1\right)},y_{i}^{\left(1\right)},x_{i}^{\left(2\right)},y_{i}^{\left(2\right)},S_{i})\right\}}_{i=1}^{N_{m}}$$

where $B^{\left(m\right)}$ denotes the complete set of bounding boxes produced by model *m*, and *N_m_*is the total number of predicted boxes for that model on a single image. Each bounding box $b_{i}^{\left(m\right)}$ consists of five elements: $\left(x_{i}^{\left(1\right)},y_{i}^{\left(1\right)}\right)$ and $\left(x_{i}^{\left(2\right)},y_{i}^{\left(2\right)}\right)$ indicates the positions of the top-left and bottom-right separately, and *S_i_*is the confidence score.

The spatial overlap between predicted bounding boxes is quantified using IoU to select candidates for WBF, as mathematically formulated in Equation 6:

(6) $$\text{IoU}(B_{i},B_{j})=\frac{\text{area}(B_{i}\cap B_{j})}{\text{area}(B_{i}\cup B_{j})}$$

where *B_i_*and *B_j_*represent two candidate boxes from the prediction set. Candidate boxes whose IoU exceeds a predefined threshold *θ* (typically *θ* = 0.5) are grouped into the same cluster $C_{k}$.

Within each cluster, WBF computes the fused bounding box coordinates by performing a confidence-weighted average of the bounding box coordinates, with the specific calculation method shown in Equation 7:

(7) $$\begin{array}{c} x^{(1)*}=\frac{\sum_{i\in C_{k}}S_{i}\;x_{i}^{\left(1\right)}}{\sum_{i\in C_{k}}S_{i}},\;y^{(1)*}=\frac{\sum_{i\in C_{k}}S_{i}\;y_{i}^{\left(1\right)}}{\sum_{i\in C_{k}}S_{i}},\\ x^{(2)*}=\frac{\sum_{i\in C_{k}}S_{i}\;x_{i}^{\left(2\right)}}{\sum_{i\in C_{k}}S_{i}},\;y^{(2)*}=\frac{\sum_{i\in C_{k}}S_{i}\;y_{i}^{\left(2\right)}}{\sum_{i\in C_{k}}S_{i}} \end{array}$$

where $S_{i}$ refers to the confidence score assigned to the i-th bounding box in cluster cluster $C_{k}$. In Equation 8, $S^{*}\;$ represents the fused box confidence, accounting for both the constituent boxes' scores and the weights of the participating models:

(8) $$S^{*}=\frac{\sum_{i\in C_{k}}S_{i}\;W_{m(i)}}{|C_{k}|}\cdot \frac{\sum_{m\in M_{k}}W_{m}}{\sum_{m\in M}W_{m}}$$

where $W_{m(i)}$ is the weight of the model corresponding to the i-th box, $M_{k}$ denotes the set of models that participate in the fusion for cluster $C_{k}$, M is the set of all models, and $|C_{k}|$ is the number of boxes in the cluster.

Finally, the fused bounding box $B^{*}$ for cluster $C_{k}$ can be formulated as shown in Equation 9:

(9) $$B^{*}=\{x^{(1)*},y^{(1)*},x^{(2)*},y^{(2)*},S^{*}\}$$

All fused boxes generated from highly overlapping clusters constitute the pseudo-label set for the current iteration. Compared with applying NMS directly on multi-model predictions, WBF effectively integrates information from multiple models, preserves high-quality predictions, refines the accuracy and robustness of pseudo-labels, leading to better outcomes in downstream semi-supervised germination detection.

### 2.5 Feature distillation loss

Feature distillation, a type of knowledge distillation, conveys information by matching intermediate feature representations of the teacher and student models (Liu et al., 2023). In the KD-SSGD framework, although the student model and the distilled student model share the same architecture, their backbone networks differ significantly in scale. To address this, we propose FDL as a core technique. Leveraging the student model’s internal semantic features, this approach transfers knowledge to the lightweight distilled student model, improving its ability to capture spatial layouts and semantic information. Compared with knowledge transfer methods that rely solely on the final outputs (Hinton et al., 2015), feature distillation fully exploits multi-scale semantic information from deep layers of the large model, thereby enhancing the distilled student’s ability to model complex spatial structures.

Specifically, we extract intermediate feature maps from the FPN layers of both the student and the distilled student models, and employ the Mean Squared Error with the Frobenius norm as the distance metric. To ensure spatial consistency, when the resolutions of the feature maps differ, the distilled student feature map $f_{ds}^{\left(i\right)}$ is resized via bilinear interpolation to obtain its aligned version ${\tilde{f}}_{ds}^{\left(i\right)}$, which matches the spatial dimensions of the corresponding student feature map $f_{s}^{\left(i\right)}$, as mathematically expressed in Equation 10:

(10) $${\tilde{f}}_{ds}^{\left(i\right)}=\text{Interp}\;(f_{ds}^{\left(i\right)},\;H_{i}^{s}\times W_{i}^{s})$$

where Interp(·) denotes bilinear interpolation, $H_{i}^{s}$ and $W_{i}^{s}$ are the height and width of the i-th student feature map, $f_{ds}^{\left(i\right)}\in {\mathbb{R}}^{C_{i}\times H_{i}^{ds}\times W_{i}^{ds}}$ is the original distilled student feature map, and ${\tilde{f}}_{ds}^{\left(i\right)}\in {\mathbb{R}}^{C_{i}\times H_{i}^{s}\times W_{i}^{s}}$ is its aligned version.

Based on the aligned feature representations, the feature distillation loss quantifies deviations of the the distilled-student model from student model, as defined in Equation 11:

(11) $$\text{KD}=\frac{1}{N_{f}}\sum_{i=1}^{N_{f}}\frac{1}{C_{i}H_{i}^{s}W_{i}^{s}}{∥f_{s}^{\left(i\right)}-{\tilde{f}}_{ds}^{\left(i\right)}∥}_{F}^{2}$$

where $C_{i}$ denotes the number of channels, while $H_{i}^{s}$ and $W_{i}^{s}$ represent the height and width of the student feature map. The Frobenius norm $∥\cdot {∥}_{F}$ measures the element-wise squared differences across all spatial locations and channels.

This loss strengthens the representational capacity of the distilled student model, enabling it to capture semantic and spatial patterns more effectively. Moreover, by being integrated into the overall training objective and jointly optimized with other supervised and unsupervised losses, FDL contributes to improving both the accuracy and the stability of pseudo-label generation, thereby enhancing the overall robustness of the framework.

### 2.6 Branch-adaptive weighting

In multi-task learning, the losses of different branches often exhibit scale discrepancies and inconsistent convergence speeds, which can adversely affect training stability and overall performance. To address this issue, previous studies have proposed dynamic weight allocation strategies. Cipolla et al (Cipolla et al., 2018). employed task uncertainty as an indicator, estimating the intrinsic noise variance of each task to adaptively adjust weights instantly. Liu et al (Liu et al., 2019). instead tracked the gradient dynamics of each loss during training to adjust task weights accordingly. Although these two approaches respectively leverage *intrinsic task noise* and *training process signals*, they can still suffer from weight adjustment lag or convergence oscillations in complex scenarios.

To enable dynamic weight adjustment for multi-branch losses, we propose a BAW mechanism. The final loss results from a weighted sum of the losses contributed by each branch as formulated in Equation 12:

(12) $$L_{\text{total}}=\sum_{i=1}^{N}{\tilde{L}}_{i}$$

where ${\tilde{L}}_{i}$ represents the weighted loss of the i-th branch. To simultaneously incorporate both branch uncertainty and training dynamics, where the weighted loss is formulated as shown in Equation 13:

(13) $${\tilde{L}}_{i}=\text{exp}(-l_{i})\cdot \text{exp }(\frac{r_{i}(t)}{T})L_{i}+\lambda_{\text{reg}}\;l_{i}$$

(14) $$l_{i}=\text{log }\sigma_{i}^{2}$$

where $L_{i}$ is the original loss of the i-th branch, $\lambda_{\text{reg}}$ is the regularization coefficient, $\sigma_{i}$ is a learnable standard deviation representing the noise level of the branch, and $l_{i}$ denotes its logarithmic transformation, as defined in Equation 14. To further adapt to the training process in real time, the rate of change in the loss for each branch is defined as shown in Equation Equation 15:

(15) $$r_{i}(t)=\frac{L_{i}(t-1)}{L_{i}(t-2)}$$

where *L_i_*(*t* − 1) and *L_i_*(*t* − 2) denote the branch losses in the two previous iterations, respectively. The temperature hyperparameter *T* controls the influence of the loss change rate on weight adjustment.

By performing joint optimization, the BAW mechanism simultaneously leverages task uncertainty and real-time training dynamics to adaptively weight the influence of individual branches on the overall loss function. This effectively mitigates instability caused by scale discrepancies and inconsistent convergence speeds, thereby improving overall model performance.

### 2.7 Implementation details

All experiments were carried out on an NVIDIA RTX 3090 GPU with 24 GB of memory. During semi-supervised training, each batch consisted of five images, using a 1:4 ratio of labeled to unlabelled data. Training was performed for 80,000 iterations. Training was carried out using AdamW, with the initial learning rate configured at 0.0002 and weight decay at 0.0001. A linear warm-up strategy was applied during the first 500 iterations with a start factor of 0.001. Subsequently, the learning rate was scheduled using the MultiStepLR policy, where it was decayed to 0.1× of its original value at the 48,000th and 60,000th iterations. The teacher model was updated using an EMA with an update interval of one iteration. For the network architecture, the distilled student branch employed a ResNet-18 backbone, while both the student and teacher branches used ResNet-50 backbones.

## 3 Results

### 3.1 Detection performance on different labeled ratios

To comprehensively evaluate the proposed KD-SSGD framework under varying levels of supervision, the evaluation was performed on the MG and TGC datasets using four labeled data ratios (1%, 2%, 5%, and 10%), simulating scenarios ranging from extremely low to moderate supervision. For an unbiased evaluation, all techniques, including the proposed KD-SSGD and representative semi-supervised baselines (Mean Teacher, Soft Teacher, and Dense Teacher)—employed the same Faster R-CNN detector, allowing direct comparison with the fully supervised counterpart.

On the MG dataset (**Table 3**), KD-SSGD consistently outperforms the fully supervised baseline and demonstrates significant advantages over semi-supervised methods such as Dense Teacher, Soft Teacher, and Mean Teacher. With 1% labeled data, our method achieves a mAP of 47.0%, surpassing fully supervised Faster R-CNN (35.6%) by 11.4% while outperforming Dense Teacher (45.0%), Soft Teacher (45.1%), and Mean Teacher (41.9%). Its mAP@50 and mAP@75 reached 64.3% and 57.7%, respectively, demonstrating strong performance in both overall and high-IoU object detection. With 2% labeled data, KD-SSGD’s mAP improves to 59.3%, maintaining its lead over the fully supervised baseline (47.1%) while remaining competitive against Dense Teacher (56.3%), Soft Teacher (57.5%), and Mean Teacher (58.6%). For 5% and 10% labeled data ratios, our method achieves mAP of 62.8% and 65.1%, respectively. At 5%, it slightly outperforms Dense Teacher, while at 10%, it falls slightly below Dense Teacher but still surpasses other semi-supervised methods, maintaining high performance on mAP@75.

**Table 3:** Detection performance comparison on the MG validation set under different labeled ratios.

Labeling ratio	Method	mAP (Δ)	mAP@50 (Δ)	mAP@75 (Δ)
1%-labeled	Faster R-CNN	35.6	56.5	39.2
	Mean Teacher	41.9 (+6.3)	57.6 (+1.1)	50.1 (+10.9)
	Soft Teacher	45.1 (+9.5)	58.6 (+2.1)	55.2 (+16.0)
	Dense Teacher	45.0 (+9.4)	63.1 (+6.6)	45.0 (+5.8)
	KD-SSGD (Ours)	47.0 (+11.4)	64.3 (+7.8)	57.7 (+18.5)
2%-labeled	Faster R-CNN	47.1	66.4	56.7
	Mean Teacher	58.6 (+11.5)	74.4 (+8.0)	71.8 (+15.1)
	Soft Teacher	57.5 (+10.4)	72.7 (+6.3)	69.9 (+13.2)
	Dense Teacher	56.3 (+9.2)	72.3 (+5.9)	60.1 (+3.4)
	KD-SSGD (Ours)	59.3 (+12.2)	74.7 (+8.3)	72.4 (+15.7)
5%-labeled	Faster R-CNN	57.0	74.4	69.6
	Mean Teacher	60.9 (+3.9)	76.1 (+1.7)	73.4 (+3.8)
	Soft Teacher	61.2 (+4.2)	76.9 (+2.5)	73.6 (+4.0)
	Dense Teacher	62.1 (+5.1)	79.3 (+4.9)	74.0 (+4.4)
	KD-SSGD (Ours)	62.8 (+5.8)	78.1 (+3.7)	75.5 (+5.9)
10%-labeled	Faster R-CNN	61.7	78.0	74.0
	Mean Teacher	61.9 (+0.2)	77.3 (-0.7)	74.4 (+0.4)
	Soft Teacher	63.9 (+2.2)	79.5 (+1.5)	76.4 (+2.4)
	Dense Teacher	66.1 (+4.4)	82.3 (+4.3)	77.2 (+3.2)
	KD-SSGD (Ours)	65.1 (+3.4)	80.4 (+2.4)	77.4 (+3.4)

On the TGC dataset (**Table 4**), KD-SSGD consistently outperforms the fully supervised baseline and demonstrates competitive performance against other semi-supervised methods, including Dense Teacher. With 1% labeled data, KD-SSGD achieves a mAP of 73.3%, surpassing fully supervised Faster R-CNN (62.0%) by 11.3% and slightly outperforming Dense Teacher (71.4%), Mean Teacher (71.9%), and Soft Teacher (71.4%). Its corresponding mAP@50 and mAP@75 were 91.1% and 87.6%, respectively. At a 2% annotation rate, KD-SSGD attains an mAP of 75.3%, exceeding the fully supervised baseline (67.1%) and exhibiting comparable or superior performance to mainstream semi-supervised detection methods, such as Dense Teacher (74.3%), Soft Teacher (74.7%), and Mean Teacher (74.5%). For annotation rates of 5% and 10%, KD-SSGD achieves mAP of 75.6% and 76.1%, respectively, maintaining a clear advantage over Faster R-CNN (72.7% and 73.7%). While its performance on mAP@50 and mAP@75 metrics is slightly below Dense Teacher, it remains stable and efficient overall.

**Table 4:** Detection performance comparison on the TGC validation set under different labeled ratios.

Labeling ratio	Method	mAP (Δ)	mAP@50 (Δ)	mAP@75 (Δ)
1%-labeled	Faster R-CNN	62.0	82.9	74.8
	Mean Teacher	71.9 (+9.9)	90.4 (+7.5)	86.5 (+11.7)
	Soft Teacher	71.4 (+9.4)	89.5 (+6.6)	85.3 (+10.5)
	Dense Teacher	71.4 (+9.4)	90.6 (+7.7)	85.6 (+10.8)
	KD-SSGD (Ours)	73.3 (+11.3)	91.1 (+8.2)	87.6 (+12.8)
2%-labeled	Faster R-CNN	67.1	85.8	81.6
	Mean Teacher	74.5 (+7.4)	92.5 (+6.7)	89.4 (+7.8)
	Soft Teacher	74.7 (+7.6)	92.8 (+7.0)	89.4 (+7.8)
	Dense Teacher	74.3 (+7.2)	92.6 (+6.8)	88.9 (+7.3)
	KD-SSGD (Ours)	75.3 (+8.2)	92.8 (+7.0)	89.5 (+7.9)
5%-labeled	Faster R-CNN	72.7	90.6	87.2
	Mean Teacher	74.6 (+1.9)	92.8 (+2.2)	89.5 (+2.3)
	Soft Teacher	75.4 (+2.7)	93.0 (+2.4)	89.9 (+2.7)
	Dense Teacher	75.2 (+2.5)	93.6 (+3.0)	90.1 (+2.9)
	KD-SSGD (Ours)	75.6 (+2.9)	93.0 (+2.4)	90.0 (+2.8)
10%-labeled	Faster R-CNN	73.7	91.2	87.4
	Mean Teacher	74.5 (+0.8)	92.3 (+1.1)	89.6 (+2.2)
	Soft Teacher	75.1 (+1.4)	93.0 (+1.8)	89.9 (+2.5)
	Dense Teacher	75.8 (+2.1)	94.5 (+3.3)	91.0 (+3.6)
	KD-SSGD (Ours)	76.1 (+2.4)	93.3 (+2.1)	90.4 (+3.0)

Overall, KD-SSGD demonstrates robustness, effective utilization of unlabeled data, and stable high precision detection performance, with the most pronounced benefits observed in low-label regimes across both datasets.

### 3.2 Ablation study

An ablation study was performed on the MG dataset using a semi-supervised configuration with 10% labeled data to assess the contributions of WBF, FDL, and BAW to the KD-SSGD framework.

For the Teacher branch, as shown in **Table 5**, incorporating the WBF module increased mAP, mAP@50, and mAP@75 by 1.2%, 1.3%, and 1.2%, respectively. This demonstrates that WBF effectively improves pseudo-label localization by integrating confidence information from both Teacher and Student predictions, maintaining stable gains even at high IoU thresholds and validating the “retain rather than discard” pseudo-box strategy proposed in Section 3.2. Adding the FDL module further improved mAP to 64.3%, with additional gains of 1.1%–2.3% across all metrics, indicating that feature distillation enhances the Student model’s perception of deep multi-scale semantic information and bridges the feature extraction gap between Teacher and Student. Incorporating the BAW mechanism led to the best overall performance, with mAP reaching 65.1% and cumulative improvements of 3.2%, 3.1%, and 3.1% over the baseline. BAW dynamically adjusts the weights of multiple branch losses, adaptively balancing convergence rates and feature scale differences between Teacher and Student, significantly enhancing training stability and final detection accuracy. The Teacher model results are compared against the mean-teacher baseline to provide a reference for performance.

**Table 5:** Ablation study of WBF, FDL, and BAW in KD-SSGD on the MG dataset (10% labeled data) —Teacher branch results.

Method	WBF	FDL	BAW	mAP (Δ)	mAP@50 (Δ)	mAP@75 (Δ)
Baseline (Mean Teacher)	–	–	–	61.9	77.3	74.3
KD-SSGD (Teacher)	✓	–	–	63.1 (+1.2)	78.6 (+1.3)	75.5 (+1.2)
	✓	✓	–	64.3 (+2.4)	79.6 (+2.3)	76.8 (+2.5)
	✓	✓	✓	65.1 (+3.2)	80.4 (+3.1)	77.4 (+3.1)

For the Distill Student branch, all ablation experiments were conducted based on WBF (see **Table 6**). Starting from WBF-only results (mAP: 53.5%, mAP@50: 67.5%, mAP@75: 64.5%), adding FDL improved the metrics to 55.7%, 69.8%, and 67.1%, with gains of 2.2%, 2.3%, and 2.6%, respectively, demonstrating that feature distillation effectively enhances the semantic perception of the lightweight Student model. Further combining BAW increased mAP to 60.2%, mAP@50 to 74.7%, and mAP@75 to 71.8%, achieving cumulative improvements of 6.7%, 7.2%, and 7.3% compared to WBF-only. These results demonstrate the effectiveness and stability of multi-branch collaborative training for the Distill Student model.

**Table 6:** Ablation study of WBF, FDL, and BAW in KD-SSGD on the MG dataset (10% labeled data) —Distill Student branch results.

Method	WBF	FDL	BAW	mAP (Δ)	mAP@50 (Δ)	mAP@75 (Δ)
KD-SSGD (Distill Student)	✓	–	–	53.5	67.5	64.5
	✓	✓	–	55.7 (+2.2)	69.8 (+2.3)	67.1 (+2.6)
	✓	✓	✓	60.2 (+6.7)	74.7 (+7.2)	71.8 (+7.3)

**Figure 3:**
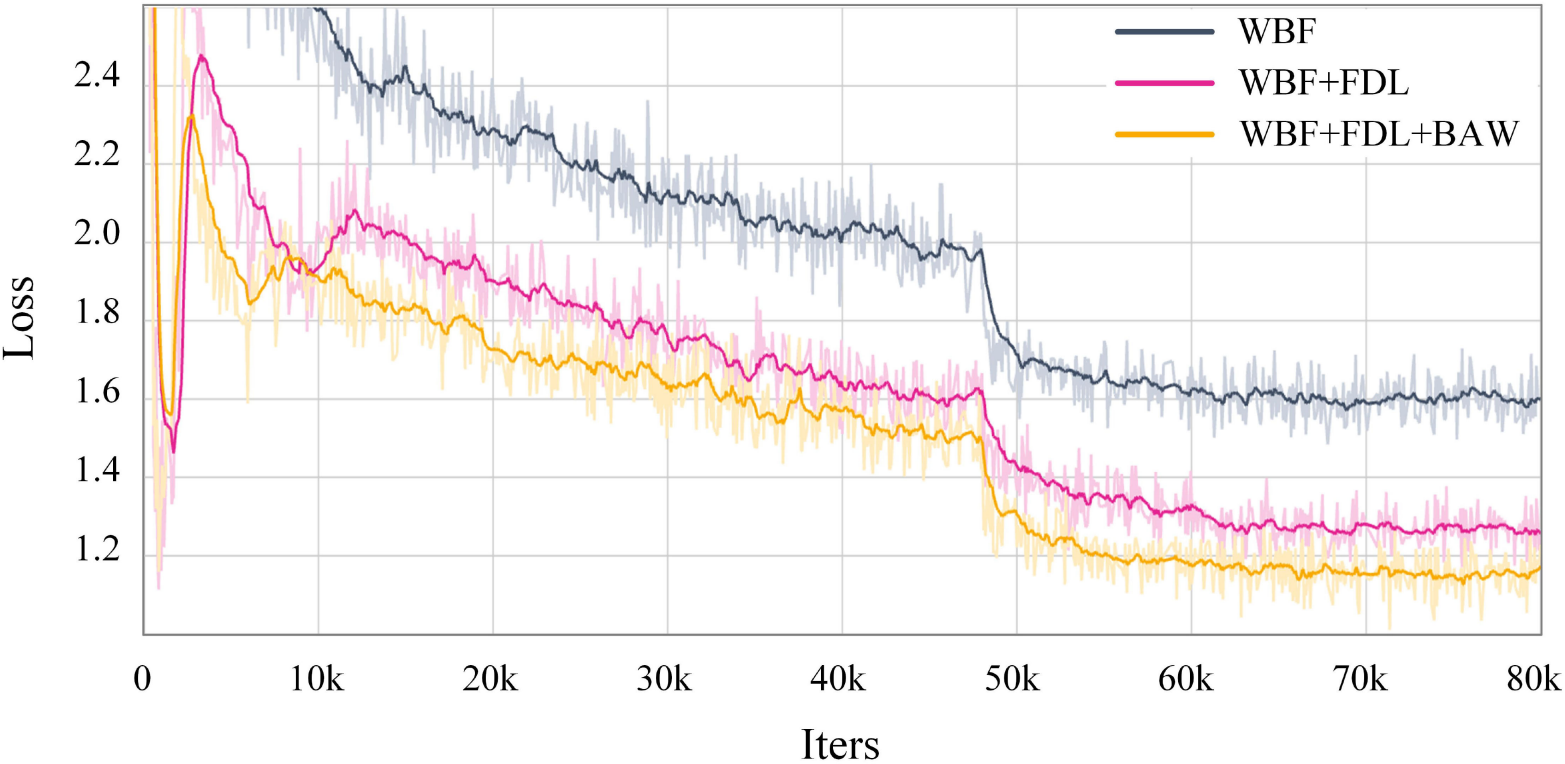
Total training loss curves of the ablation study on the MG dataset under the semi-supervised setting with 10% labeled data. Based on WBF, the total loss gradually decreased as FDL and BAW were added, validating the effectiveness of each component in the KD-SSGD.

As shown in **Figure 3**, the total training loss, based on WBF, gradually decreased as FDL and BAW were added, further validating the effectiveness of each component in the KD-SSGD. Overall, WBF, FDL, and BAW synergistically contributed to improved pseudo-label quality, feature transfer, and training optimization within the KD-SSGD, ensuring consistent performance gains and enhanced generalization in semi-supervised object detection.

**Figure 4:**
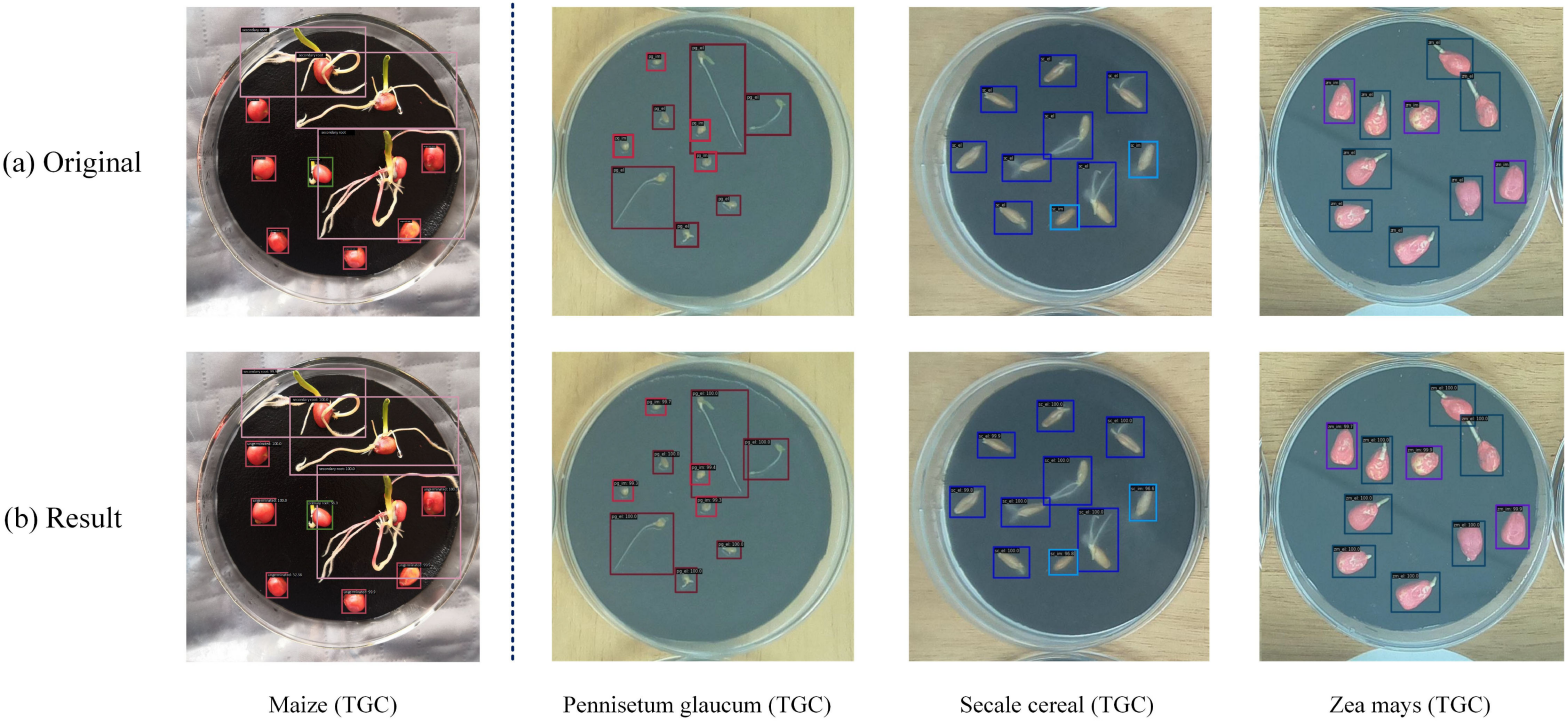
Visualization of germination detection on the MG and TGC datasets using the proposed KDSSGD method. **(a)** Ground-truth annotations and **(b)** detection results under the semi-supervised setting with 10% labeled data, where bounding boxes indicate the predicted outputs.

In summary, WBF, FDL, and BAW each played a critical role in both Teacher and Distill Student branches. WBF improved pseudo-label localization, FDL enhanced feature transfer for the lightweight student model, and BAW optimized multi-branch training. For both datasets, the germination detection results are illustrated in **Figure 4**. These empirical findings indicate that the proposed semi-supervised seed germination detection method consistently demonstrates robust performance under different ratios of labeled data. Collectively, the three mechanisms establish a stable and efficient semi-supervised learning system, supporting continuous improvements in detection accuracy and generalization capability.

## 4 Discussion and conclusion

This study proposes KD-SSGD, a semi-supervised germination detection framework enhanced by knowledge distillation, designed to address the challenges of limited annotations and complex seed germination morphology in agricultural scenarios. The framework introduces a lightweight distilled student branch together with three core modules: WBF, FDL, and BAW. These components sequentially enhance pseudo-label quality, enable effective multi-scale knowledge transfer, and stabilize multi-branch optimization. Comprehensive experiments on the Maize-Germ (MG) and Three Grain Crop (TGC) datasets demonstrate that KD-SSGD consistently outperforms mainstream semi-supervised methods under various annotation ratios, while maintaining high accuracy and robustness even with extremely sparse labels (1% and 2%). These results highlight its potential to substantially reduce annotation costs and provide a scalable solution for intelligent agricultural perception. Moreover, as shown in **Table 7**, training efficiency comparisons indicate that KD-SSGD achieves superior accuracy while incurring only minor additional computational overhead compared to baseline methods, thereby confirming its computational feasibility and practicality for real-world deployment.

**Table 7:** Comparison of training time and per-iteration cost for different methods on the MG and TGC datasets.

Dataset	Method	Training time (h:min)	Avg. iteration time (s)
MG	Mean Teacher	17:24	0.78
	Soft Teacher	18:21	0.83
	Dense Teacher	18:13	0.82
	KD-SSGD	19:02	0.86
TGC	Mean Teacher	11:47	0.53
	Soft Teacher	15:48	0.71
	Dense Teacher	15:04	0.67
	KD-SSGD	15:11	0.68

Results are reported under a single GPU (1 × RTX 3090) and a single training task with 10% labeled data. All methods were trained for 80,000 iterations.

Despite these advantages, KD-SSGD still faces several challenges. The current framework models only single-frame information, which limits its capacity to track the temporal dynamics of germination. It also relies solely on RGB imagery, which remains sensitive to illumination changes and occlusion in complex field conditions. In addition, the framework requires training from scratch on each dataset, leading to considerable computational cost and limited scalability.

Future work will therefore focus on three promising directions. First, incorporating temporal modeling could enable the framework to capture germination dynamics by leveraging growth trajectories and radicle emergence patterns, thereby yielding more biologically meaningful predictions (Adak et al., 2024). Second, the integration of multimodal fusion (e.g., hyperspectral or multispectral data) may provide complementary cues to improve robustness under diverse environmental conditions (Yang et al., 2025b). Third, applying transfer learning can enhance adaptability across different crops and imaging environments, while simultaneously improving detection accuracy and reducing computational cost (Hossen et al., 2025). Collectively, these extensions will further strengthen KD-SSGD’s accuracy, stability, and applicability in real-world agricultural scenarios.

## Data Availability

The original contributions presented in the study are included in the article/supplementary material. Further inquiries can be directed to the corresponding author.
